# Circulating plasma circular RNAs as novel diagnostic biomarkers for congenital heart disease in children

**DOI:** 10.1002/jcla.22998

**Published:** 2019-08-20

**Authors:** Jinhuan Wu, Jiaqing Li, Heng liu, Jiangwen Yin, Mengjie zhang, Zhangbin Yu, Hongjun Miao

**Affiliations:** ^1^ Department of Emergency Medicine Children's Hospital of Nanjing Medical University Nanjing China; ^2^ Department of Anesthesiology Children's Hospital of Nanjing Medical University Nanjing China; ^3^ Department of Pediatrics Obstetrics and Gynecology Hospital Affiliated to Nanjing Medical University Nanjing China

**Keywords:** biomarkers, circular RNAs, congenital heart diseases, diagnosis

## Abstract

**Objective:**

The diagnostic value of circulating circular RNAs (circRNAs) has received more and more attention. However, little has been reported about their potential in the diagnosis of congenital heart diseases (CHD). In this study, we explored differential expression of circRNAs from children with CHD to evaluate their potential as clinical biomarkers.

**Methods:**

We established a discovery cohort (four CHD cases; four matched healthy controls) and a validation cohort (40 CHD cases; 40 matched healthy controls). Microarray expression analysis was performed on the discovery set to identify candidate circRNAs. Candidates were further validated in the validation set. The diagnostic accuracy of circRNAs was determined by receiver operating characteristic (ROC) analysis. Gene ontology (GO), pathway, and network analysis were performed to predict a network of circRNA/miRNA and target mRNAs related to CHD.

**Results:**

The top seven significantly differentially expressed CHD‐associated circRNAs were validated by RT‐PCR as follows: hsa_circRNA_004183, hsa_circRNA_079265, hsa_circRNA_105039, hsa_circRNA_404686, hsa_circRNA_101050, hsa_circRNA_100787, and hsa_circRNA_101328. Three significantly down‐regulated circRNAs (hsa_circRNA_004183, hsa_circRNA_079265, and hsa_circRNA_105039) were identified with area under curve (AUC) values of 0.758, 0.809, and 0.907, respectively; the combination had an AUC of 0.965. An interaction network was constructed by 43 circRNAs, 9 miRNAs, and 29 mRNAs, which involved in heart development.

**Conclusions:**

We identified three circRNAs under‐expressed in plasma from children with CHD. These circRNAs may be crucial in the development of CHD and may serve as novel non‐invasive biomarkers for the diagnosis of CHD in children.

## 1 INTRODUCTION

Congenital heart disease (CHD) is one of the most common causes of death among children under five years of age in China.^1^ The causes of cardiac‐related mortality also include deaths from pulmonary hypertension, bacterial endocarditis, and congestive heart failure. However, complex CHD is the main cause of early death in children under five years old.^2^ Approximately 8 in 1000 children suffer from CHD. Despite the advances in diagnosis, surgery, and interventional therapy, the mortality rate of children with CHD in China remains high.^3^ Furthermore, our knowledge regarding the causes of CHD remains limited.^4^ Early diagnosis of CHD improved disease prognosis,^5^ and the improved diagnosis of childhood CHD will be effective in reducing CHD mortality among children. High‐efficiency and specific clinical biomarkers are urgently desired for childhood CHD.

Ultrasound echocardiography was employed as a screening tool for CHD, although cardiac abnormalities could be undiagnosed during routine examination. The lack of standardization of ultrasound examinations resulted in variance in diagnosis results from clinic to clinic.^6^ Many factors can influence the accuracy of sonographer investigations for the detection of CHD, such as the experience of operators, the quality of the ultrasound equipment, the lesion type, as well as the departmental policies and guidelines at individual institutions. Extraordinary diagnostic precision leads to low morbidity and mortality, which characterizes the state of the art in the clinical management of CHD. As to clinician caring for a child with CHD, it is critical to determine whether there is an underlying genetic element associated with the disease. Recent advancements in molecular biology techniques have resulted in greater elucidation of the molecular mechanisms of the heart formation, including bone morphogenetic protein 4,^7^ miRNAs,^8^, ^9^ lncRNAs,^10^ and peptides located within the functional domains of precursor proteins identified essential for heart development.^11^ However, little is known about the role of circular RNAs (circRNAs) in the pathology of CHD.

Circular RNAs are a large class of non‐coding RNAs that exist ubiquitously in the cytoplasm of eukaryotic cells.^12^, ^13^ These endogenous RNAs are characterized by a stable structure and exhibit tissue‐specific expression patterns.^14^ Compared with linear RNAs, circRNAs uniquely undergo non‐canonical splicing without a free 3′ or 5′ end.^15^, ^16^ CircRNAs were proposed to function as miRNA sponges and were believed to antagonize miRNA‐dependent gene regulation, thus contributing substantially to the competing endogenous RNA network.^17^ CircRNAs were widely involved in physiological/pathological processes, including nervous system disorders,^18^, ^19^, ^20^ cancer,^21^, ^22^ and preeclampsia.^23^ CircRNAs are becoming important biological molecules for understanding the mechanisms of disease and exploring biomarkers for disease diagnosis and treatment. Werfel et al^24^ observed extensive differential expression of circRNAs between neonatal and adult rat hearts, which was closely related to heart disease.^25^, ^26^

In this study, we performed microarray analysis to identify circRNAs from the plasma of children with CHD. We identified three circRNAs under‐expressed in plasma from children with CHD compared with healthy controls. These circRNAs may serve as novel biomarkers for the diagnosis of CHD in children.

## 2 MATERIAL AND METHODS

### 2.1 Study design and patient samples

In this study, paired cases and controls were used to confirm the predictive utility of circulating circRNAs in the diagnosis of CHD by echocardiography. Between September 2015 and March 2016, cases of children undergoing surgery for congenital heart disease were collected at Nanjing Children's Hospital. Children with high blood pressure, cancer, diabetes, nervous system disease, Down syndrome, and other congenital diseases or deformities were excluded. A total of 40 cases with congenital heart disease were selected for this study. All 40 cases of CHD were either ventricular septal defect (VSD) or atrial septal defect (ASD). Forty healthy children were included as a control group. In order to reduce heterogeneity, controls were matched with cases on the basis of age and gender. Samples were collected with written consent and ethics board approval.

The study was divided into two phases: biomarker discovery and biomarker validation. In the biomarker discovery phase (Phase I), expression of circRNA in plasma samples from four randomly selected CHD cases and four matched controls were evaluated by Arraystar microarray to identify circRNAs differentially expressed in children with CHD compared with healthy children. Gene ontology (GO) analysis and signal pathway analysis were performed to evaluate the function of differentially expressed circRNAs. The circRNAs exhibiting the most significant differential expression were chosen as candidate biomarkers for further large scale sample validation. In the biomarker validation phase (Phase II), expression of these candidate biomarkers was evaluated by quantitative reverse transcription‐polymerase chain reaction (qRT‐PCR) from 40 CHD cases and 40 healthy controls. Based on the results of this phase, receiver operating characteristic (ROC) curve‐based risk assessment analysis was conducted on candidate circRNAs to assess the sensitivity and specificity of these biomarkers in plasma to predict CHD in children.

### 2.2 Plasma preparation and RNA extraction

Two milliliters of blood from each child in the CHD and control groups was collected in an EDTA‐anticoagulant tube and centrifuged at 1000 *g* for 10 min. Plasma was stored at −80°C in 1.5 mL RNase‐free microcentrifuge tubes for further use. Total RNA, including circRNAs, was extracted from the plasma using the TRIZOL LS kit (Life Technologies) according to the manufacturer's instruction. The purity and concentration of the total RNA was determined using a NanoDrop1000 (Thermo Scientific). Total RNA was reverse‐transcribed into cDNA using the RevertAid First Strand cDNA Synthesis Kit (Thermo Scientific). cDNA was preserved at −80°C for later use. The handling and storage of samples was identical to reduce potential intra‐ and inter‐assay error.

### 2.3 Arraystar microarray

In phase I of the experiment, circRNAs circulating in the plasma of four CHD cases and four healthy controls were screened using the Arraystar gene chip detection system. Briefly, total RNAs were digested with Rnase R (Epicenter, Inc) to remove linear RNAs and enrich circular RNAs. Then, the enriched circular RNAs were amplified and transcribed into the fluorescent cRNA utilizing a random priming method (Arraystar Super RNA Labeling Kit; Arraystar). The labeled cRNAs were hybridized onto the Arraystar Human circRNA Array V2.0 (8x15K, Arraystar). After having washed the slides, the arrays were scanned by the Agilent Scanner G2505C. Differentially expressed circRNAs between the two groups were screened using *P*‐value/FDP, and the differential expressions of circRNAs between two samples were screened through fold change. Finally, GO analysis and signal pathway analysis were performed, and circRNAs displaying significant changes between CHD cases and healthy controls were selected for further examination.

### 2.4 Reverse transcription and quantitative PCR

Total RNA was reverse‐transcribed to cDNA with a TaqMan reverse transcription kit. A 20 μL reaction mixture consisted of 16 μL nuclease‐free water, 1μg total RNA, and 4 μL of 5× PrimeScript^®^ RT Master Mix. Reverse transcription was initiated at 37°C for 15 min, terminated by incubation at 85°C for 15 seconds, and held at 4°C.

Subsequent real‐time PCR was performed in triplicate for each plasma sample from CHD cases and healthy controls. All primers were designed by Primer 5 software and synthesized by Shanghai GENEray Biotech. The circRNAs Assay IDs are shown in Table 1. For the final qPCR reaction, 1 μL of synthesized cDNA was mixed with 3 μL of diethylpyrocarbonate (DEPC)‐treated water, 5 μL of SYBR Green master mix, and 0.5 μL of each forward and reverse primers. The mixture was incubated at 50°C for 2 minutes, 95°C for 10 minutes, followed by 40 cycles of 95°C for 15 seconds and 55°C for 1 minutes. The quantitative PCR results were normalized to the reference gene GAPDH. Probe specificity to amplify a single PCR product was confirmed by melting curve analysis and gel electrophoresis. The relative expression of target circRNAs was determined using the comparative cycle threshold (Ct) method (2^−∆Ct^), where ∆Ct = Ct_sample_−Ct_β‐actin_.^27^, ^28^

**Table 1:** Sequence of primers for the internal reference gene and circular RNAs (circRNAs)

	5′‐3′ (sense)	5′‐3′ (antisense)
GAPDH	CATGAGAAGTATGACAACAGCCT	AGTCCTTCCACGATACCAAAGTCC
hsa_circRNA_101328	ACGGCGTCACCAACCTACC	TCGCCTGCTGTCCAAATGA
hsa_circRNA_105039	GGAGAATGAGGACGGCACTT	CCTTCGGGATCCGTCACTT
hsa_circRNA_004183	CGTCCATTCCACGAGGTTCT	GCCTCTGACGCAGGGTTTC
hsa_circRNA_079265	TGGGAGTGGGTGGAGGCAG	GCCTCGTCGCCCACATAGG
hsa_circRNA_100787	TGGGACAAGCAAACCTTTA	GTGGGGCTCTGGTACTGAA
hsa_circRNA_101050	CCCGGGAAGAGCTGATGAG	GTGGCAGTCTGGTTTTGGC
hsa_circRNA_404686	GCGTCCATTCCTTTGATT	TTCCCGTCTTTACCAGCA

### 2.5 Differentially expressed circRNA host genes enrichment analysis

To study the main function of differentially expressed circRNA host genes, we assessed down‐regulated genes by GO analysis and KEGG pathway enrichment analysis. Results were visualized, and a comprehensive database analysis (DAVID) was used to incorporate GO analysis and KEGG pathway enrichment analysis. Significant differential expression was considered as *P*‐value < .05 and fold change ≥ 2.

### 2.6 Statistical analysis

All statistical analysis were performed with SPSS software version 22.0 (SPSS, Inc). Differences in the demographic, clinical characteristics, and the relative expression levels of circRNAs between two groups were estimated by the Student's *t* test. A *P‐*value < .05 was considered statistically significant, and all statistics were two‐sided. The efficiency of circRNAs as a diagnostic tool for CHD in children was evaluated with ROC curves. The sensitivity and specificity of each circRNA were assessed by analysis of the area under the ROC curve (AUC); 95% confidence intervals (CI) are provided.

## 3 RESULTS

### 3.1 Subject characteristics

A total of 80 participants were enrolled in this study, including 40 children with CHD and 40 healthy children as controls. There were 30 cases with VSD and 10 cases with ASD. The diagnosis of CHD was confirmed by echocardiography. As shown in Table 2, No differences were observed between two groups regarding age, RBC, HGB, ALT/AST,Cr (*P* > .05). However, LDH, CK, CK‐MB were significantly different between two groups (*P* < .05).

**Table 2:** General features of the children in the congenital heart diseases (CHD) and healthy control groups

	CHD (mean ± SD)	Control (mean ± SD)	*P*‐value
Ages (months)	33.82 ± 15.98	34.25 ± 18.12	.92
RBC (×10^12^/L)	4.42 ± 0.72	4.19 ± 0.58	.11
HGB (g/L)	122.43 ± 12.45	123.65 ± 13.02	.67
ALT/AST	0.51 ± 0.17	0.56 ± 0.19	.33
LDH (U/L)	315.50 ± 91.66	216.05 ± 51.28	<.05
CK (U/L)	167.65 ± 162.46	89.83 ± 38.97	<.05
CK‐MB (U/L)	38.53 ± 12.89	19.78 ± 4.4	<.05
Cr (μmmol/L)	30.73 ± 6.24	30.18 ± 5.50	.68

### 3.2 Arraystar microarray analysis

**Figure 1:**
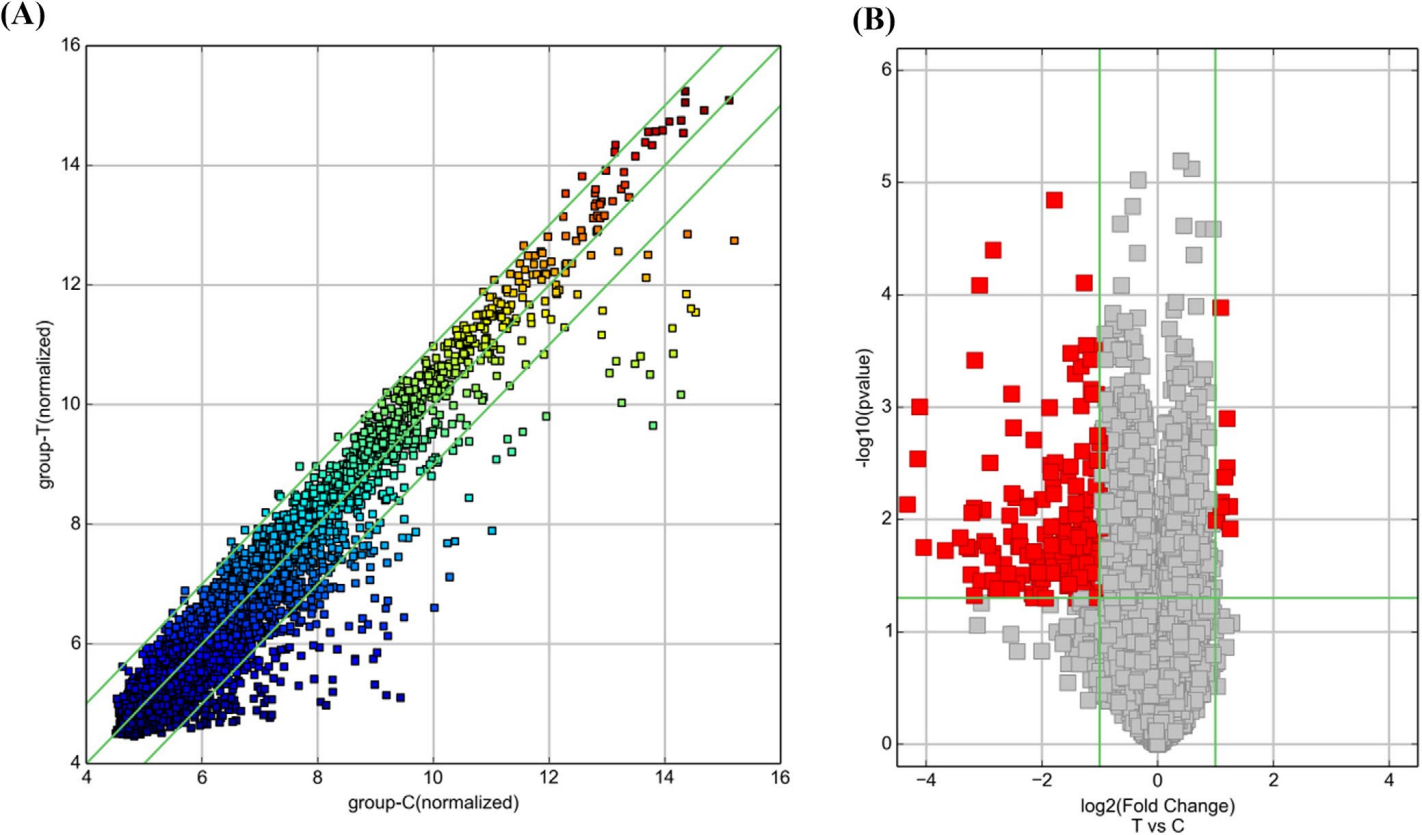
Differential expression of circular RNAs (circRNAs). (A) The scatter plot is a visualization method used for assessing the variation in circRNA expression. The values corresponding to the *X*‐axis and *Y*‐axis in the scatter plot are the normalized signal values of the samples (log2 scaled). The green lines indicate fold changes. The circRNAs above the top green line and below the bottom green line indicate more than 2.0‐fold changes between the two groups. (B) Volcano plots were constructed using fold‐change values and *P*‐values. The vertical lines correspond to 2.0‐fold over‐ and under‐expression between two groups, and the horizontal line represents a threshold *P*‐value. The red points in the plot represent significantly differentially expressed circRNAs (*P* < .05)

Expression profiling of circRNAs identified 8543 circulating circRNAs differentially expressed in the plasma of children with CHD compared with healthy children (Figure 1A). Ten circRNAs were significantly over‐expressed (fold change ≥ 2.0), and 157 circRNAs were significantly under‐expressed in the CHD group (Figure 1B).

### 3.3 Gene ontology and pathway analysis

**Figure 2:**
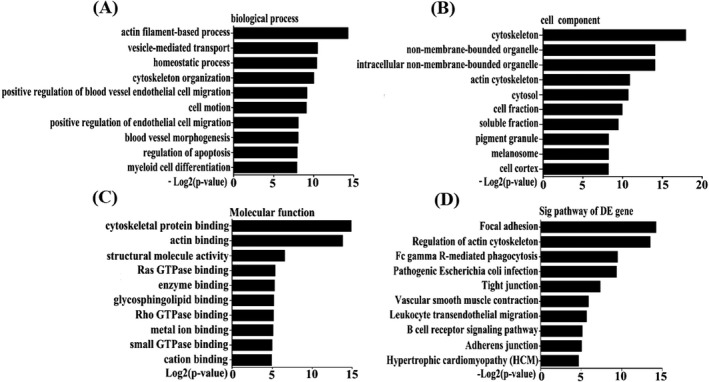
Gene ontology (GO) and subcellular locations of differentially expressed circular RNAs (circRNAs). GO analysis enrichment of (A) biological processes, (B) cellular components (C) molecular functions; (D) Pathways corresponding to under‐expressed circRNA transcripts. The bar graph shows the top enrichment score value of the significantly enriched pathway

Gene ontology analysis was performed to describe the circRNAs in terms of the biological processes, cellular components, and molecular functions that these circRNAs may interact with or regulate. Fisher's exact test was used to determine whether there was more overlap between the list of differentially expressed circRNAs and the GO annotation list than that would be expected by chance. The p‐value denoted the significance of GO term enrichment in the differentially expressed genes. The lower the p‐value, the more significant the GO term (*P* < .05 was considered significant). The most highly enriched GOs terms associated with down‐regulated circRNA transcripts among biological processes, cellular components, and molecular functions were shown in Figure 2A‐C.

One of the most enriched signaling pathways was the focal adhesion kinase (FAK) pathway (Figure 2D), which involved in heart chamber development.^29^ The most enriched signaling pathway was the “adherens junction” pathway.

### 3.4 Biomarker discovery

The aim of this study was to identify circRNAs in circulating plasma of children that could serve as biomarkers for predicting CHD. Differential expression of circRNAs between four children with CHD and four matched healthy controls were performed using the Arraystar gene chip. The average expression levels and the fold change of circRNAs were analyzed. Based on the GO analysis, seven candidate circRNAs were identified that might be closely relate to CHD. To further assess these circRNAs, primers were designed to analyze their expression by qPCR (Table 1).

Three significantly under‐expressed circRNAs were selected for further large sample validation (hsa_circRNA_004183, hsa_circRNA_079265, hsa_circRNA_105039; *P* < .05). The results were shown in Table 3.

**Table 3:** Differential expression of circular RNAs (circRNAs) in plasma between two groups

circRNA	Control (mean ± SD)	CHD (mean ± SD)	*P*‐value
hsa_circRNA_004183	4.02 ± 2.61	7.19 ± 2.43	＜.05
hsa_circRNA_079265	2.09 ± 1.37	4.23 ± 2.59	＜.05
hsa_circRNA_105039	1.56 ± 1.19	5.63 ± 2.54	＜.05
hsa_circRNA_404686	4.25 ± 2.57	4.92 ± 2.51	.24
hsa_circRNA_101050	3.47 ± 2.48	3.81 ± 2.51	.55
hsa_circRNA_100787	5.68 ± 2.22	6.43 ± 3.39	.25
hsa_circRNA_101328	5.74 ± 2.96	4.86 ± 2.94	.19

### 3.5 Validation of circRNAs as biomarkers for CHD

**Figure 3:**
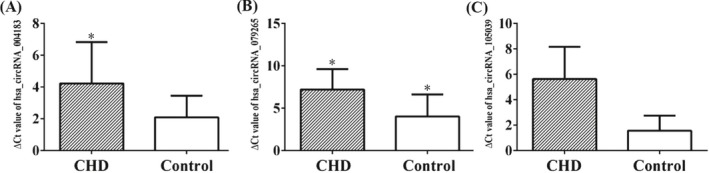
The expression levels of circular RNAs (circRNAs) in cases with congenital heart diseases (CHD) and healthy controls. Expression histogram (A) plasma hsa_circRNA_004183, (B) plasma hsa_circRNA_079265, and (C) plasma hsa_circRNA_105039. *P < .05

The three candidate circRNAs were validated by RT‐PCR with samples obtained from 40 children with typical CHD and 40 healthy controls. The result of the qPCR was normalized to the reference gene GAPDH. Three of the seven candidate circRNAs (hsa_circRNA_004183, hsa_circRNA_079265, and hsa_circRNA_105039) were significantly differentially expressed in CDH cases compared with the healthy controls (*P*‐values < .05, Table 3), whereas expression levels of the other four candidate circRNAs were not significantly different between the two groups (*P*‐values of 0.24, 0.55, 0.25, and 0.19, respectively; Table 3 and Figure 3).

### 3.6 Clinical utility of plasma circRNA levels in predicting CHD in children

**Figure 4:**
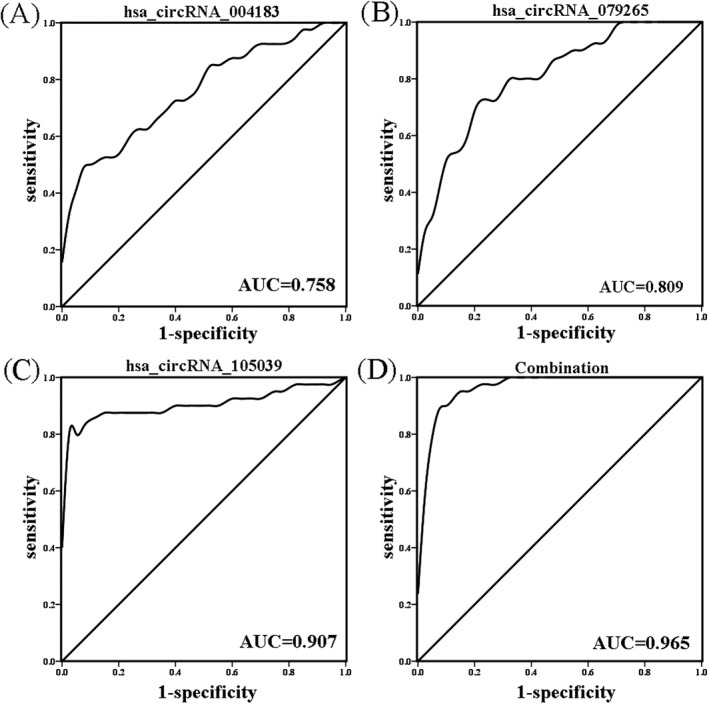
Receiver operating characteristic (ROC) curves for three candidate circular RNAs (circRNAs). (A) hsa_circRNA_004183, (B) hsa_circRNA_079265, (C) hsa_circRNA_105039; (D) receiver operating characteristic (ROC) curve for all three circRNAs combined

The sensitivity and specificity of the individual circulating plasma circRNAs in the prediction of CHD were assessed using ROC curve analysis. The result agreed with the ROC curves (Table 4 and Figure 4). The area under the ROC curve for the three under‐expressed circRNAs individually (hsa_circRNA_004183, hsa_circRNA_079265, hsa_circRNA_105039) were 0.758, 0.809, and 0.907, respectively. Considering the three circRNAs as a combined biomarker for CHD, the area under the ROC curve was 0.965 (Table 4). These results suggested that expression of the three circRNAs might be an effective biomarker to predict CHD in children.

**Table 4:** The sensitivity, specificity, and area under curve (AUC) of receiver operating characteristic (ROC) curves of three circular RNAs (circRNAs) individually and combined

	Sensitivity	Specificity	AUC	95% CI
hsa_circRNA_004183	0.5	0.95	0.758	0.716‐0.902
hsa_circRNA_079265	0.725	0.8	0.809	0.552‐0.790
hsa_circRNA_105039	0.8	1	0.907	0.831‐0.983
Combination	0.9	0.925	0.965	0.930‐1.0

### 3.7 Prediction of the circRNA/miRNA and target genes related to CHD

**Figure 5:**
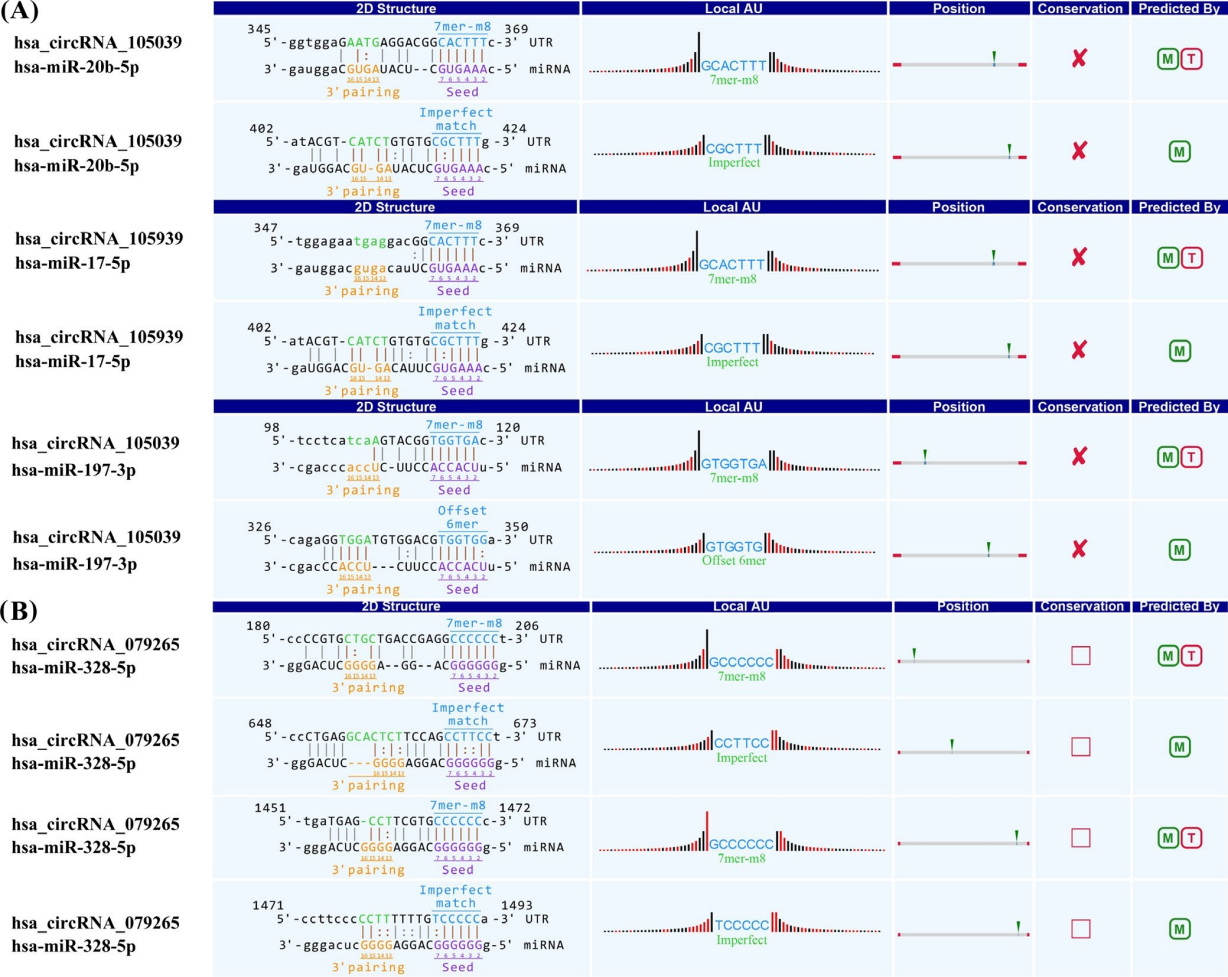
Potential microRNAs targeted by circular RNAs (circRNAs). (A) The potential miRNA targets of hsa_circ_105039 include hsa‐miR‐20b‐5p, hsa‐miR‐17‐5p, and hsa‐miR‐197‐3p. (B) The potential miRNA target of hsa_circ_079265 is hsa‐miR‐328‐5p

Recent studies have reported that circRNAs functioned as miRNA sponges to regulate miRNA expression and miRNA‐dependent gene regulation.^14^, ^17^, ^30^ The interaction of circRNAs with disease‐associated miRNAs indicated that circular RNAs were important in disease regulation.^17^, ^19^, ^30^ To find the potential miRNA targets, we investigated the ability of miRNA to bind with candidate circRNAs. The circRNA/miRNA interaction was predicted by Arraystar's custom miRNA target prediction software based on TargetScan & miRanda. Two confirmed circRNAs (hsa_circ_105039, hsa_circ_079265) were annotated in detail using circRNA/miRNA interaction information. The potential miRNA targets of hsa_circ_105039 included hsa‐miR‐20b‐5p, hsa‐miR‐17‐5p, and hsa‐miR‐197‐3p (Figure 5A). For hsa_circ_079265, the potential miRNA target was hsa‐miR‐328‐5p (Figure 5B).

**Figure 6:**
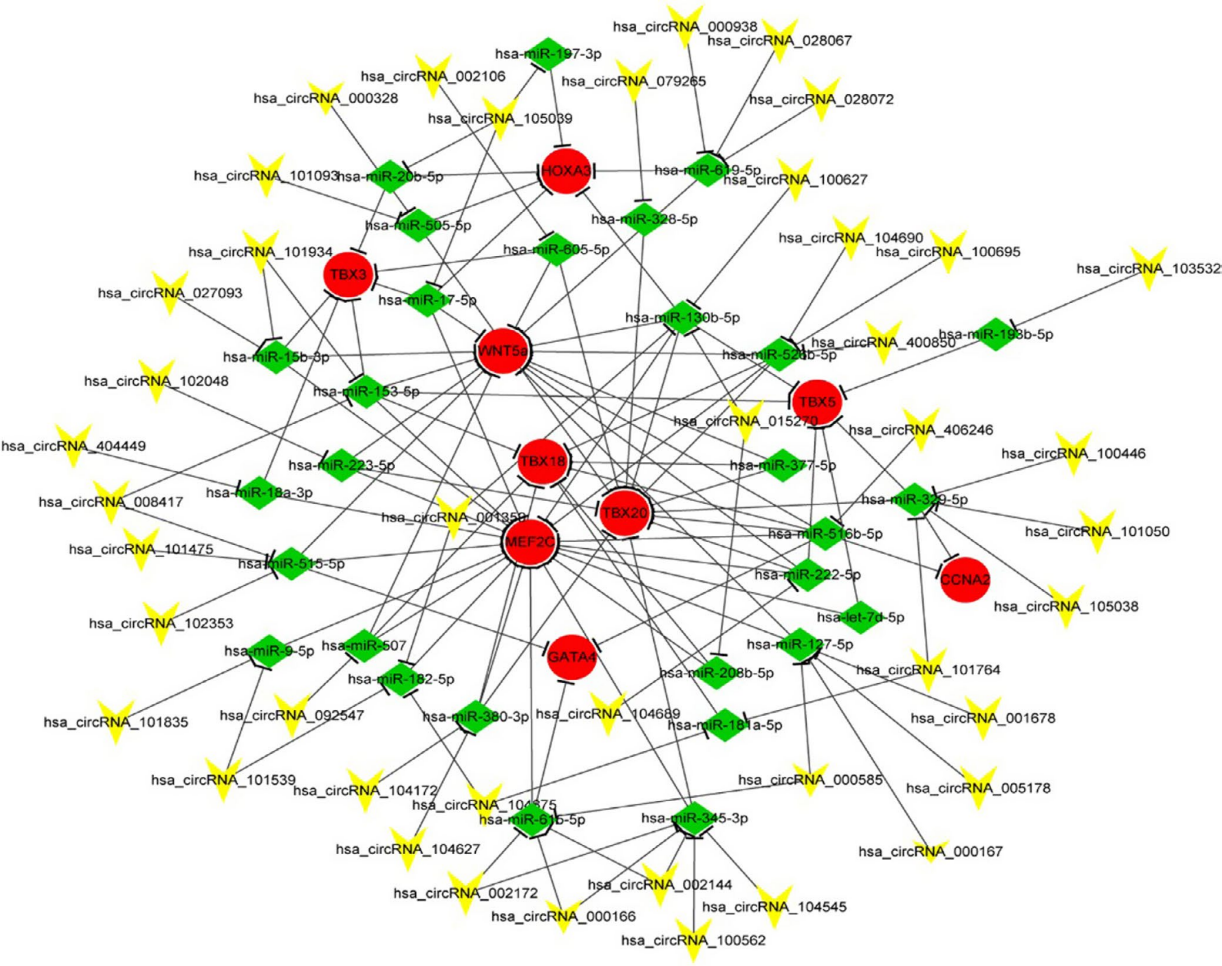
Circular RNAs (circRNAs)‐miRNA‐mRNA interaction network. The network consists of 9 nodes (genes). In the circRNA‐miRNA‐mRNA network, the inverted triangles represent circRNAs, circles represent mRNAs, and rhombuses represent miRNAs. An interaction between two factors is represented by one edge

Gene co‐expression networks were used to predict the circRNA targets. A network was constructed by miRNAs commonly predicted to bind circRNAs and mRNAs. We selected CHD‐related mRNAs and predicted their binding miRNAs. Through merging the common miRNA targets, we constructed a network of circRNAs‐miRNAs‐mRNAs with a total of 43 circRNAs, 9 mRNAs, and 29 miRNAs. (Figure 6). Each gene corresponded to a node, and two genes were connected by a string. The network indicated the tight correlation and regulatory relationship of the genes, miRNAs, and circRNAs. The potential mRNA targets for miR‐17‐5p included wnt5a, MEF2C, TBX3, HOXA3. The potential mRNA targets for miR‐20b‐5p included TBX3, and HOXA3. For miR‐193‐3p and miR‐24‐3p, the target gene was HOXA3 and WNT5a, respectively.

## 4 DISCUSSION

The mammalian heart is a complex organ, and its formation is strictly regulated by many genes, such as those encoding transcription factors, epigenetic factors, miRNAs, and signaling pathways. All of these genes established complex regulatory networks for cardiac development.^31^, ^32^ Some studies have focused on the epigenetic regulation of CDH pathogenesis and potential targets for therapy, including microRNAs and long non‐coding RNAs (lncRNAs).^9^, ^10^ However, the involvement of circRNAs in heart development and CDH pathogenesis remains largely unknown. In this study, for the first time, we profiled circRNA expression in plasma of children with CHD and identified a number of circRNAs that are aberrantly expressed in children with CHD compared with healthy children.

Circular RNAs (circRNAs) are a type of non‐coding RNAs (ncRNAs) produced in eukaryotic cells during post‐transcriptional processes. Recent studies have shown that many exonic transcripts can form circRNAs through non‐linear reverse splicing or gene rearrangement. CircRNAs potentially regulate wide aspects of cellular physiology, including miRNA binding, translational regulation, protein interactions, and even protein translation. CircRNAs were widely expressed in human cells, and their expression levels could be higher by 10‐fold or more compared with their linear isomers.^33^ CircRNAs contain highly conserved sequences and possess good stability in mammalian cells.^20^ These properties provided circRNAs with potential as stable biomarkers and therapeutic targets.^27^ CircRNAs can function as miRNA sponge, which may alleviate the inhibitory effects of miRNAs on target molecules, thereby regulating gene expression levels. It has been reported that the antisense sequence for the cerebellar degeneration‐related protein1 transcript (CDR1as) is a representative molecular sponge. CDR1as contained approximately 74 binding sites for miR‐7, and CDR1as over‐expression down‐regulated miR‐7 expression levels.^20^, ^34^ The repression of miR‐7 function by CDR1as revealed a new therapeutic strategy for treatment of Alzheimer's disease. Circular RNA products were discovered to originate from the ANRIL locus, and the causal variants at 9p21.3 regulated INK4/ARF expression and influenced atherosclerotic vascular disease risk by modulating ANRIL expression and structure.^35^ Khan MA et al^36^ reported that several circRNAs expressed from the titin locus gene were dynamically regulated in dilated cardiomyopathy. Furthermore, they showed that RBM20 was crucial for the formation of a subset of circRNAs originating from the I‐band of the titin gene. Vausort M et al^25^ reported the expression of the circRNA MICRA, when measured at reperfusion in peripheral blood samples of patients with acute myocardial infarction, predicted left ventricular dysfunction after 3‐4 months. A number of other studies showed that circRNAs were closely related to the heart and cardiovascular system diseases, suggesting that circRNAs might be useful as diagnostic or therapeutic biomarkers.

In our study, we identified three circRNAs that were significantly under‐expressed in children with CHD compared with healthy ones. The validation results agreed with the ROC curves. To examine the potential biological consequences of the under‐expression of these circRNAs, we constructed a network including circRNAs and mRNAs and a circRNA‐miRNA‐mRNA interaction network. These two networks suggested the potential associations between circRNAs and their target genes. Additionally, the networks provided an important reference value for studying the interaction of other differentially expressed circRNAs with their potential targets.^37^ In this study, we predicted the interaction of circRNA/miRNA and the target genes related to CHD. We found that the potential miRNA targets for hsa_circ_105039 included miR‐20b‐5p, miR‐17‐5p, miR‐197‐3p, and miR‐24‐3p. Zhu et al reported that miR‐20b were critical in apoptosis, differentiation, and mitochondrial function of P19 cells. They further suggested that miR‐20b might not only represent a novel therapeutic target for congenital heart diseases but also provide new insights into the mechanisms of cardiac diseases.^38^ The potential mRNA targets for miR‐17‐5p included wnt5a, MEF2C, TBX3, and HOXA3. Wnt5a were produced in the OFT by cells originating from the pharyngeal mesoderm signals to adjacent CNC cells during the formation of the aortopulmonary septum.^39^ Mef2c could regulate the expression of cardiac extracellular matrix protein.^40^

However, we only compared the circRNA expression in 40 pairs of patients. The cohort is not large enough to get a definite conclusion. Therefore, more samples should be collected in the future. Further cell and animal model experiments should be conducted to increase comprehension of the detailed mechanism and specific functions of circRNAs in CHD.

In summary, we identified and validated three circRNAs which might act as potential non‐invasive biomarkers for the diagnosis of children with CHD. These findings may provide potential targets for the future treatment of CHD and novel insights into the mechanisms underlying the biology of CHD.

## CONFLICTS OF INTEREST

The authors declare no conflicts of interest regarding the publication of this article.

## ETHICAL APPROVAL

Ethical approval was given by the medical ethics committee of the IEC of Nanjing Children's Hospital Affiliated to Nanjing Medical University with the following reference number:201601003‐1.
